# Migrating in a Warming World: A Deep Learning Approach to Predict Pan‐American Seasonal Shifts in the Monarch Butterfly Niche

**DOI:** 10.1111/gcb.70805

**Published:** 2026-03-27

**Authors:** Chiara Vanalli, Robin Zbinden, Nina van Tiel, Devis Tuia

**Affiliations:** ^1^ Environmental Computational Science and Earth Observation Laboratory École Polytechnique Fédérale de Lausanne Sion Switzerland

**Keywords:** citizen science, climate change scenarios, deep learning, ecosystem health, future projections, model explainability

## Abstract

Climate change is driving biodiversity loss, disrupting ecosystem functioning, and altering species distributions. Migratory species, whose range varies across seasons depending on specific climatic conditions, are particularly sensitive to environmental changes and serve as indicators of ecosystem health. However, current species distribution models often fail to capture the temporal dynamics critical for migratory species, limiting their ability to provide accurate future range estimations. In this study, we address this gap by developing a time‐aware deep learning species distribution model for the monarch butterfly ( *Danaus plexippus* ), an iconic species for biodiversity conservation. Using monarch occurrence records across the Americas gathered from scientific and citizen science sources, we incorporate the effect of monthly climatic variables in a sequential framework. We compare the performance of our concatenated seasonal model to conventional time‐static baselines, showing not only better performance in the present, where the models have been trained and validated, but also in the past. Our findings show that climatic factors such as humidity, temperature, precipitation and cloud coverage strongly influence the ecological niche of the monarch butterfly, with notable seasonal and spatial variability. Applying our model under climate change scenarios, we predict a northwestward shift in the monarch range by the end of the XXI century, with expansion in Canada and significant contraction in California and Mexico, key sites for overwintering that also host resident monarch populations. These changes could severely impact the species' migratory cycle and population stability. Using Shapley values, an explainable AI technique, we identify the decrease in precipitation and an increase in temperature as important environmental drivers responsible for the contraction of overwintering sites. By focusing on a species of high ecological relevance through a time‐aware modeling approach, this work brings novel insights for the conservation of migratory species in the face of the challenges posed by climate change.

## Graphical abstract

Migratory species are highly sensitive to environmental changes, making them useful indicators of how ecosystems respond to global change. To address the limitations of time‐static species distribution models, which fail to capture dynamic range shifts, we develop a time‐aware deep learning model to study the monarch butterfly migration across the Americas. Beyond improving model performance, our approach links expected changes in environmental conditions to a northwestward distribution shift and highlights range contractions in California and Mexico, threatening overwintering sites and long‐term population stability.

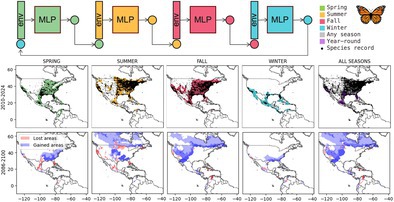

## 1 Introduction

Climate change is profoundly affecting ecosystem health by altering weather patterns and frequency of extreme events, with disruptive consequences on the ability of species to survive, reproduce, and thrive (Malhi et al. 2020; Turner et al. 2020). The ongoing pervasive decline in biodiversity serves as a clear signal of the threat that anthropogenic pressures pose to ecosystems (Mooney et al. 2009). The degradation of ecosystems and the essential services that they provide undermines human well‐being with significant economic and societal costs (Cardinale et al. 2012; Pecl et al. 2017). These challenges highlight the urgent need to deepen our understanding of the link between species and the environment in which they live, as well as to develop innovative tools that can track and predict the evolving status of ecosystems in response to environmental changes. By translating these insights into actionable strategies, science‐informed conservation measures can effectively enhance the resilience of ecosystems and safeguard the diversity of the species they host.

Migratory species play a key role in ecological networks, by transporting nutrients and impacting trophic webs; therefore, providing important ecological services along their migratory route (Bauer and Hoye 2014; Wilcove and Wikelski 2008; Satterfield et al. 2020). Whether driven by survival or reproduction, migrations are an adaptive response to specific environmental conditions and are, in turn, highly impacted by environmental changes (Wilcove and Wikelski 2008; Satterfield et al. 2020; Shaw 2016; Robinson et al. 2009). Global warming has contributed to earlier migration and breeding time (Both and te Marvelde 2007), shorter migration distances (Visser et al. 2009), and loss of breeding ground (Zylstra et al. 2021) with detrimental alterations to the migration journey. Among the various migratory taxa, insects provide a particularly striking example. Insects are one of the animal groups most affected by climate change, with detected pervasive effects in terms of behavior, phenology, physiology, and distribution (Harvey et al. 2023, 2020). Therefore, understanding how migratory insect species distributions are shaped by climatic conditions and how they are expected to shift due to climate change is key for ecosystem and biodiversity conservation (Bauer and Hoye 2014; Satterfield et al. 2020). In this respect, ecological niche modeling can provide crucial insights, identifying the range occupied by a species in relation to environmental variables (Grinnell 1924; Vandermeer 1972) and its expansion, contraction, and shift due to climate change (Chen et al. 2011; Parmesan and Yohe 2003; Silva et al. 2019). However, mapping the distribution of migratory species to the environment is challenging due to the intrinsic spatial and temporal connectivity that characterizes migration: gaps in tracking individual movements throughout the entire migratory cycle and the overlook of the complex impacts of the multiple seasonal climatic factors represent the main limitations and lead to the current partial success of modeling migratory patterns in space and time at high resolution.

Recent technological advancements have brought new opportunities for species monitoring, such as the use of positional transmitters (GPS) (Kays et al. 2015; Bridge et al. 2011), radar technologies (Chapman et al. 2004), drones with cameras (Wich et al. 2021; May et al. 2024), and radiotelemetry with transmitters light enough to track even small animals like insects (Daniel Kissling et al. 2014; Knight et al. 2019). Concurrently, the growing amount of georeferenced species observations collected from the general public worldwide and shared through citizen‐science platforms represents a powerful resource for ecological and environmental sciences (Fraisl et al. 2022), but also for tackling planetary health challenges (Vanalli et al. 2024). Leveraging these novel data streams, Species Distribution Models (SDMs) have become a widely used approach to map species niches by statistically linking observed species presences/absences to environmental conditions (Elith and Leathwick 2009; Guisan and Zimmermann 2000). Despite their impressive achievements in ecological niche mapping, SDM applications to migratory species are still limited (Carbeck et al. 2022; Coxen et al. 2017). This is mainly due to the time‐static way in which environmental inputs (usually averaged over multiple decades) and species occurrence probability are treated, neglecting the temporal dynamics that are pivotal for migratory species. The diverse climatic conditions to which these species are exposed at different stages of their migratory route need to be thoroughly addressed and integrated in modeling frameworks (Ponti and Sannolo 2023; Zurell et al. 2024). Disregarding those seasonal dynamics could result in an over‐ or under‐estimation of the actual species distribution (Ponti and Sannolo 2023), hindering the model implementation under different climatic conditions from those where the model has been trained and validated (Tsiftsis et al. 2024), thus limiting their ability to draw meaningful conclusions on species ecology (Fourcade et al. 2018). Deep learning‐based SDMs (deep SDMs) have the potential to overcome the highlighted limitations, thanks to their flexibility to treat large and diverse input information (Zbinden et al. 2025; Gillespie et al. 2024), handle complex nonlinear relations, proving to be robust and effective tools to model species distribution (Botella et al. 2018; Hu et al. 2025; Zbinden et al. 2023; Brun et al. 2024) and more generally to inform biodiversity conservation (Pollock et al. 2025; Tuia et al. 2022). Additionally, advancements in eXplainable Artificial Intelligence (XAI) methods (Gunning et al. 2019; Lundberg and Lee 2017) have made it possible to transition black box models into approaches capable of identifying the relevant predictors reflecting the ecological dynamics of the target species and/or explaining observed shifts (Zbinden et al. 2025; Bourhis et al. 2023). Therefore, incorporating the temporal dimension has the potential to (i) improve the biological realism of deep SDMs, properly addressing the different role of predictors at different time periods, (ii) reduce biases in niche estimates, possibly due to the mismatch of specific environmental predictors, (iii) produce a time‐dynamic species occurrence probability that is key to follow the journey of migratory species, and (iv) enhance model robustness to predict species range alterations under rapid global change, as informed by general circulation models.

In this work, we aim to map the ecological niche of the monarch butterfly ( *Danaus plexippus* ) at a pan‐American scale and to predict future projections of the niche shifts under climate change. A key pollinator species (Ghazanfar et al. 2016) with a migratory route that extends up to 4000 km, monarchs have historically been an iconic migratory species for biodiversity conservation (Oberhauser and Solensky 2004; Gustafsson et al. 2015) and, together with other butterfly species, can be used as sentinel to monitor ecosystem health (Oostermeijer and Van Swaay 1998). Climatic factors, habitat loss, diseases, and agricultural insecticide use have threatened monarch populations in the Americas, with sharp declines over the last three decades (Thogmartin et al. 2017; James 2024). In particular, climatic changes have been identified as a major driver of monarch population dynamics with significant impacts on the breeding‐season range and population size (Zylstra et al. 2021). In 2023, the International Union for Conservation of Nature modified the conservation status of the monarch butterfly from “endangered” to “vulnerable” to extinction, after detecting a stable/positive population trend (Normile 2023), despite some criticisms from the scientific community, who showed that evidence of growing monarch population was not statistically significant (Thogmartin et al. 2020). In this study, we use large‐scale occurrence observations of monarchs, collected from both professionals and the general public, to examine the role of monthly climatic inputs for predicting their seasonal distribution across the Americas. We develop a time‐aware deep SDM that captures the temporal dynamics as seasonal sequences, and compare its performance to a baseline time‐static model. For each season, we assess the different contributions of the multiple climatic variables to the definition of species ranges, using an explainable AI approach. Last, we predict future contractions/expansions and shifts of the monarch ecological niche under different climate change scenarios, including the uncertainty generated by the multiple scenarios considered.

Evaluating how climate change shapes the ecological niche of a well‐studied species, like the monarch butterfly, with a complex ecology and migration, provides valuable insights into macroecology and biogeography of threatened species. Our approach shows clear predictive benefits, leading to a better outlining of the niche occupied by the Monarch butterfly. Our predictions for the present status and possible future ranges hold the potential of better informing science‐based conservation efforts for habitat protection and/or restoration to face the challenges posed by global warming.

## 2 Material and Methods

### 2.1 Study System: Monarch Butterfly in the Americas

The monarch butterfly ( *D. plexippus* ) is native to North and South America, where it exhibits a wide diversity of migratory behaviors (Nail et al. 2019). The two main long‐distance migratory monarch populations are: the North Eastern American population, which every year travels 4000 km in a multi‐generational manner to overwinter in mountainous forests in Mexico (Solensky 2004), and the North Western American population, which is smaller in size and also migrates in the fall, traveling up to hundreds of km to forested groves along the coast of California (Tuskes and Brower 1978; Leong et al. 2004). This migratory behavior is driven by diapause, a hormonally controlled suspended reproductive state that contributes to winter survival and to the recolonization of northern territories (Masters et al. 1988; Green and Kronforst 2019). In addition to these migratory populations, non‐migratory stable populations of monarchs have been documented in southern California (Satterfield et al. 2016; Urquhart et al. 1970), southern Florida (Brower 1961), the Gulf Coast (Howard et al. 2010), and Mexico (Pfeiler et al. 2017). Seasonal climate variability is the main driver of monarch migration (Green and Kronforst 2019; Fisher et al. 2024) and recent changes in climate related to global warming have been impacting monarch butterfly dynamics and distribution (Zylstra et al. 2021). The diversity of the monarch migratory behaviors, with seasonal and year‐round suitable habitat across the Americas makes studying and modeling climate change impacts challenging. In addition to the Americas, *D. plexippus* also inhabits different areas of the world (i.e., New Zeland, Australia and some islands of south western Europe; Nail et al. 2019), but for the purpose of this study we focus on the main populations in the Americas within the spatial domain that spans from −10° to 65° of latitude and from −130° to −38° of longitude.

### 2.2 Datasets: Species Occurrence and Climatic Predictors

We used presence‐only data of *D. plexippus* with georeferenced coordinates available on the Global Biodiversity Information Facility (GBIF) (Gbif.org 2024) from 2010 to 2024 to train, validate, test, and compare performance of the models described (data random split of 80% for training, 10% for validation, and 10% for testing). We considered observations recorded in (i) March, April, and May belong to the spring season, (ii) June, July, and August to the summer season, (iii) September, October, and November to the fall season, and (iv) December, January, and February to the winter season, following the seasonal labeling of the boreal hemisphere. We estimated the year‐round niche for resident monarch populations from seasonal occurrence data, that is, where at least one monarch occurrence has been recorded for each of the four seasons at a specific location, and with at least one adjacent spatial cell that belongs to the “year‐round niche.” The latter condition allows the removal of potential museum and/or tropical greenhouse specimens. The choice of the selected 15‐year time span is justified by the temporal coverage of observations with available seasonal information (Figure A1), as well as an appropriate time scale to examine the effects of climatic variables on the ecological niche of monarchs. In addition, we use *D. plexippus* presence‐only data from 1990 to 2004 that lack seasonal information (i.e., the observations do not include the temporal detail about the month of observation, see Figure A1). Given the lack of temporal information, we validate our simulations for the period 1990–2004 only at the yearly level and analyze the seasonal trends only qualitatively.

The abiotic variables used in our deepSDM are: minimum (*tasmin*), mean (*tasmean*) and maximum (*tasmax*) temperature, specific (*huss*) and relative (*hurs*) humidity, evaporation (*evapsbl*), downwelling shortwave (*rsds*) and longwave radiation (*rlds*), wind speed (*sfcwind*), precipitation (*pr*), cloud coverage (*clt*) (Table A1). The selection of these variables is supported by previous research on butterfly ecology (Zylstra et al. 2021; Bourhis et al. 2023; Kuussaari et al. 2016; Bryant et al. 2002) and by the objective of capturing the full complexity of climate and its changes. The abiotic variables are available from the Coupled Model Intercomparison Project Phase 6 (CNRM‐CM6‐1‐HR model) at a spatial resolution of $∼\left(0.5\times 0.5\right)$ degrees, for the past, present and future climate under different Shared Socioeconomic Pathways (SSPs) of the Intergovernmental Panel of Climate Change (Copernicus Climate Change Service (C3S) Climate Data Store (CDS) 2021). Climatic data were downloaded at a monthly climatic resolution and averaged over the considered 15‐year temporal horizon, therefore providing data for each month averaged over the entire time period. Specifically, we analyze a more sustainable scenario, SSP1‐2.6, an intermediate emission scenario, SSP2‐4.5, and a fossil‐fuel oriented scenario, SSP5‐8.5, over a 15‐year time period at the middle of the century, from 2046 to 2060 (Figure A5), and at the end of the century, from 2086 to 2100 (results in the main manuscript).

### 2.3 Seasonal Species Distribution Model

**FIGURE 1:**
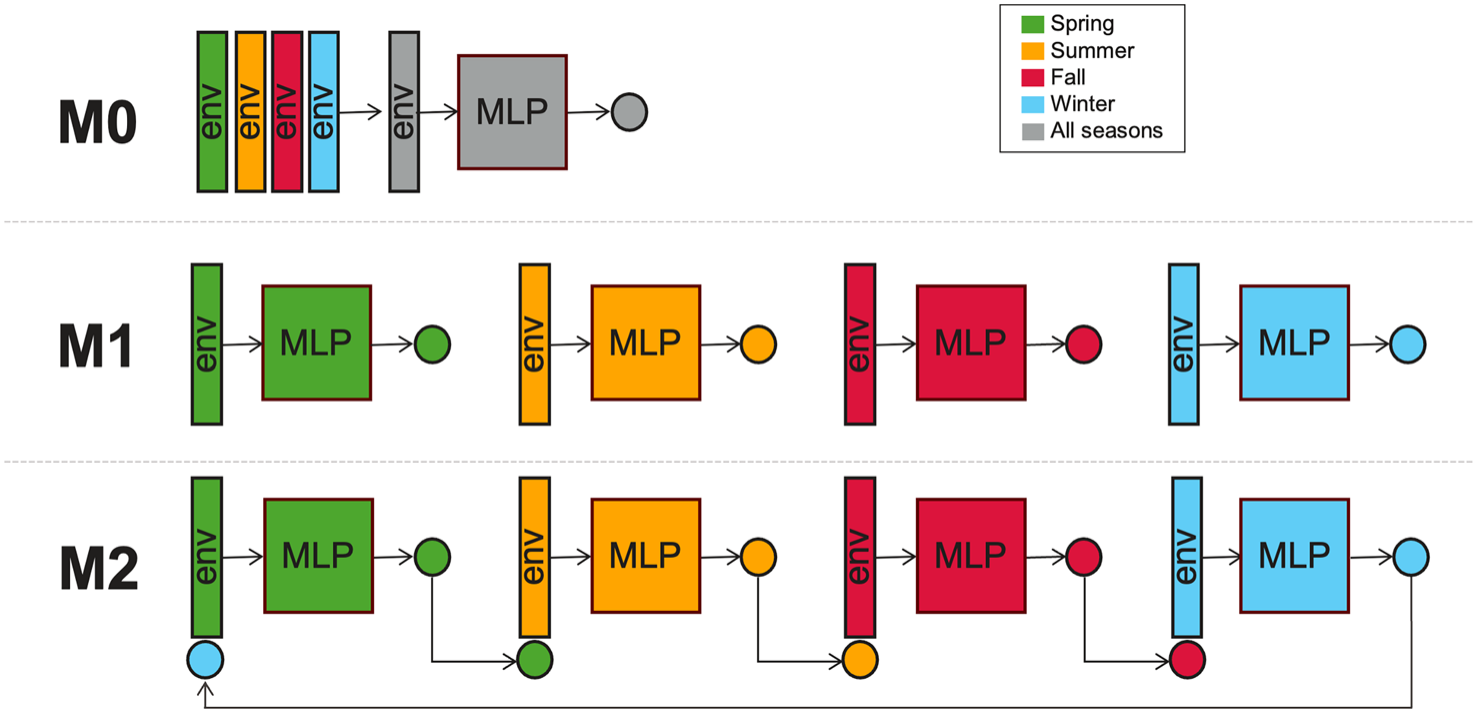
Time‐static (M0), seasonal independent (M1), and seasonal concatenated (M2) analyzed species distribution models. Monthly environmental predictors (env, Table A1) are used as inputs to Multi‐Layer Perceptrons (MLP) with monarch occurrence probability as output node. Spring season is represented in green, summer in orange, fall in red and winter in cyan, while M0 is represented in gray because it takes as input annual average predictors and produces the probability of monarch occurrence in a time‐independent manner.

Extensive modeling efforts have been made toward the understanding of the *D. plexippus* ecological niche (Fisher et al. 2024; Batalden et al. 2014; Zipkin et al. 2012) as well as toward the prediction of its future range shifts (Oberhauser and Peterson 2003; Momeni‐Dehaghi et al. 2024; Zylstra et al. 2022). Despite the recognized importance of incorporating seasonality (Ponti and Sannolo 2023; Zylstra et al. 2022), current SDMs for the monarch butterfly often focus on a restricted spatial domain (Zylstra et al. 2022; Kass et al. 2020; Neupane et al. 2022), on a specific time interval of the yearly migration cycle (Batalden et al. 2014; Zipkin et al. 2012) and/or on only a few climatic predictors, usually temperature and precipitation (Zylstra et al. 2022; Neupane et al. 2022). However, a more holistic framework that includes multiple climatic variables across the entire spatial domain of its migratory route may enable a better understanding of climate change impacts upon the monarch butterfly niche. Therefore, we incorporate the described 11 climatic predictors (Table A1) across the pan‐American continental scale as inputs to a series of Multi‐Layer Perceptrons, artificial neural networks previously implemented in species distribution modeling (Zbinden et al. 2023; Park and Lek 2016). Specifically, we compare three different modeling settings (Figure 1): (i) a time‐static model (M0) that takes as input annual average predictors across the study period (total of 11 predictors) and produces the probability of monarch occurrence in a time‐independent manner, (ii) seasonal independent models (M1) that incorporate monthly climatic predictors within the considered season (each season being composed of 3 months, this leads to a total of 3 × 11 predictors for each season) and produce the seasonal probability of monarch occurrence and (iii) seasonal concatenated models (M2) that considers as inputs, in addition to the monthly climatic predictors, the estimated probability of occurrence of the previous season, therefore propagating niche information across seasons. The seasonal models are concatenated and run sequentially and we constrained this chain of models to the three previous seasons (e.g., winter probability is estimated starting from the previous spring season). We input an extra dummy starting value that is set to 0 if the considered season is the start of the concatenation or to 1, otherwise. Overall, this setting corresponds to a total of 3 × 11 + 2 = 35 predictors for each season, where the two extra variables are the occurrence probability predicted for the preceding season and the dummy variable, respectively. The same architecture is kept across the four seasons, with two hidden layers of 40 neurons each and one output node, corresponding to the *D. plexippus* occurrence probability for the season considered. As nonlinearities, ReLU activation functions are implemented throughout the architecture, and a final sigmoidal transformation is employed to predict probability values between 0 and 1. We generate 10,000 background points (Whitford et al. 2024) in a buffer of 100 km around the convex hull defined by species observations to characterize the environmental conditions against which presences are contrasted. These are used together with presence data to train, validate, and test the M0, M1, and M2 models. Models were trained with the cross‐entropy loss for binary classification and Adam optimizer (Kingma and Ba 2014), with a learning rate of 0.01 and a maximum of 250 epochs. For M2, we provide a pseudoalgorithm in the Supplementary Infromation (Algorithm 1). At each epoch, for a randomly chosen starting season, s=1, with the starting input set to 0, $d_{1}=0$, and the previous season probabilities to 0.5, $p_{0}=0.5$, a training step is performed (forward pass, loss computation, backward pass, and parameter update). Then, for the remaining three seasons, the previous seasonal models are used sequentially to infer input probabilities up to the season under consideration, and a training step is performed on each of these seasons, with the starting input variable set to 1. To avoid potential overfitting issues, for each modeling framework (M0, M1, and M2), we apply early stopping by saving the model weights from the epoch with the lowest cross‐entropy loss evaluated on the validation set. Specifically for M2, we evaluate the global cross‐entropy loss of the validation set, computed as a sum of seasonal losses inversely weighted by each season's sample size to ensure equal consideration of each season. We assess the model performance on the test set computing the area under the ROC curve (AUC_ROC_), the area under the precision‐recall curve (AUC_PR_), and the true skill statistic (TSS). Since the latter is a threshold‐dependent metric that requires binary model outputs, we used the validation dataset to find the optimal thresholds that maximize TSS. During the time period 2010–2024, the global (i.e., any season) model performance is computed by averaging the four seasonal performances. In this same time period, we examined the performance of the Maximum Entropy method (MaxEnt), a common machine learning approach to model species distribution, using linear, hinge and product transformations of time‐static features (Phillips et al. 2006). Instead, given that no seasonal information is available for past observations (1990–2004), only a time‐static performance is evaluated. In this period, to compare occurrence probabilities generated with different seasonal models with contrasting binarization thresholds for the global TSS, we rescale the seasonal probabilities according to a 0.5 threshold: $p_{\text{rescaled},i}=p_{i}-\left({\mathrm{th}}_{i}-0.5\right)$, where *th*_*i*_ is the season‐specific probability threshold for binarization, estimated from the validation set. We compare the spatial overlapping between the estimated monarch suitability niche of the different models (Figure A3), and a spatial‐explicit accuracy comparison across the different biomes that characterize North and Central America (Dinerstein et al. 2017), which contain at least 100 occurrences (Figure A4).

To examine the contribution of each climatic predictor in defining the monarch butterfly niche, we consider the Shapley values (Shapley 1953), an XAI method (Zbinden et al. 2025; Lundberg and Lee 2017). For each considered feature and analyzed season, we calculate Shapley values, offering a measure of variable importance to the species occurrence, together with the Pearson correlation between Shapley and feature values, whose sign and magnitude represent the overall directionality effect of the focal feature on the species probability of occurrence (Bourhis et al. 2023). Despite providing general information on the feature effects, the latter method is a simplification which assumes linear relationships between feature values and Shapley values, although this is not necessarily the case. Last, we assess the difference in Shapley values between the future scenarios and the present situation to examine how changes in specific climatic predictors impact the occurrence of the monarch butterfly. While we calculate Shapley values for all the considered environmental predictors, we explore the spatial distribution of selected well‐studied variables, such as precipitation and temperature for specific seasons.

## 3 Results

### 3.1 Model Comparison and Data Fitting

**FIGURE 2:**
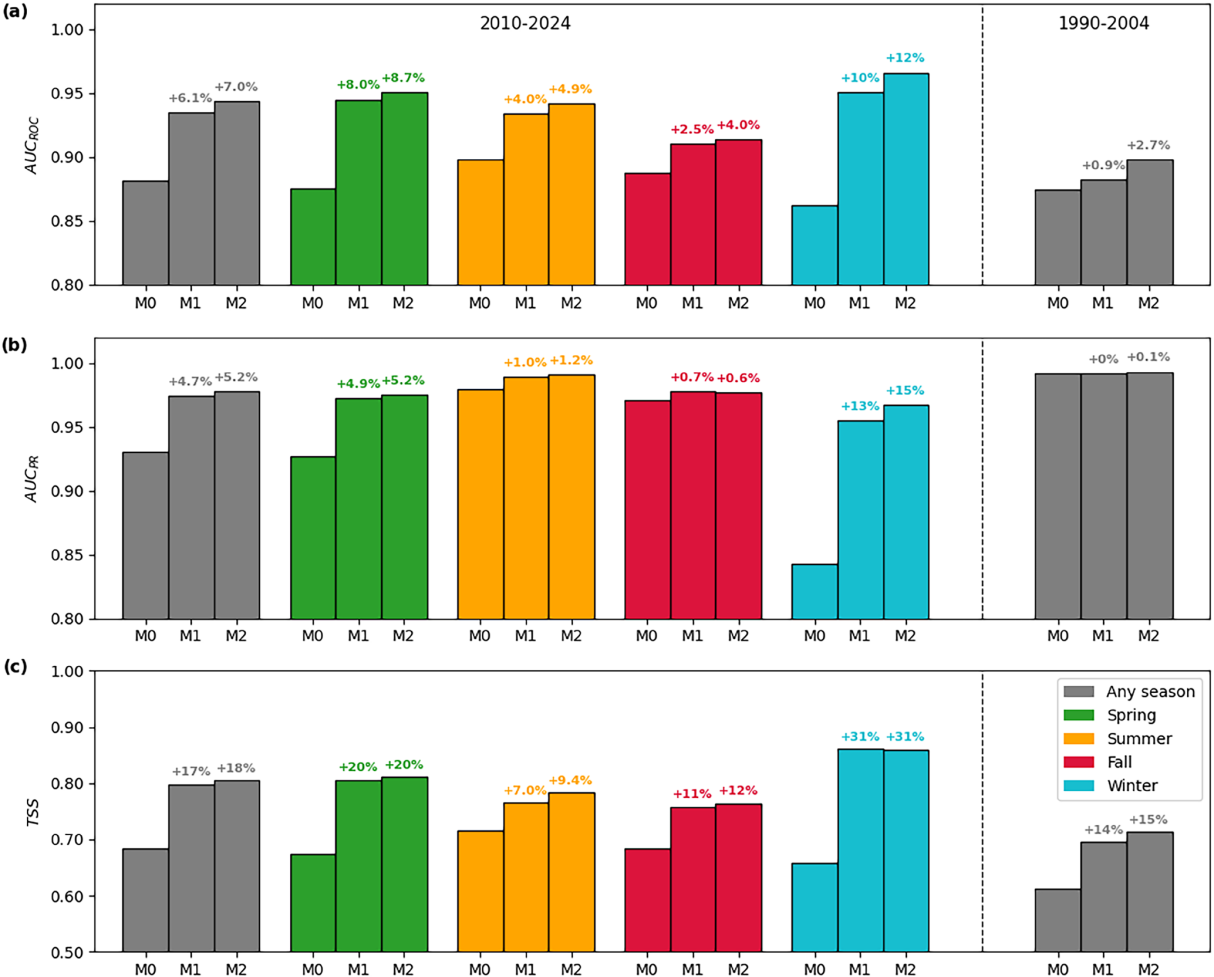
Global (any season in gray) and seasonal model performance (spring in green, summer in orange, fall in red, winter in cyan) comparison of the Area Under the ROC Curve (AUC_ROC_, a), the Area Under the Precision‐Recall Curve (AUC_PR_, b), and the True Skill Statistic (TSS, c) using the test dataset of 2010–2024 (left) and of 1990–2004 (right). M0 represents the time‐static model, M1 the seasonal independent model, and M2 the seasonal concatenated model, respectively. The percentage increase in model performance compared to the time‐static model M0 is reported on top of each bar. Full numerical results are reported in Tables A2 and A3.

We compare the performance of the time‐static model (M0), seasonal independent (M1), and concatenated models (M2) in the present, for which we have seasonal labels (2010–2024, Figure 2 and Tables A2 and A4) and in the past, for which no seasonal labels were available (1990–2004, Figure 2 and Table A3). The time‐static baseline shows good global performance, with an AUC_ROC_ of 0.881, an AUC_PR_ of 0.930, and a TSS of 0.683, confirming the important role that climatic predictors play in shaping the distribution of monarch butterflies (Zylstra et al. 2021). A comparable model performance is achieved by MaxEnt with time‐static environmental inputs, suggesting that both analyzed approaches (i.e., MLPs and MaxEnt) are able to represent well the environmental niche of the monarch butterfly (Table A4). Compared to M0, seasonal MLPs achieve higher model performance across seasons. This is even more evident for seasons with lower sample size (Figure A1), such as spring and winter, for which M0 achieves AUC_ROC_ values lower than 0.880 (Figure 2). Importantly, M0 shows a weaker performance in accurately modeling the winter distribution, a key task to identify potential overwintering sites that are essential to population preservation and migration. Deploying independent seasonal models (M1) partially solves this issue, with a greater increase in model performance in winter (+10.3% AUC_ROC_; +30.7% TSS) than in summer (+1.0 AUC_ROC_; +7.0% TSS) compared to M0. This gain in model accuracy, given by the integration of monthly climatic predictors, is an indicator of the importance of seasonality for migratory species like the monarch butterfly. Compared to M1, the seasonal concatenated model (M2) presents just one additional input (i.e., the probability of occurrence of the previous season), together with the binary starting input variable, but still results in an increase in the model performance. This increase is consistent across seasons.

When these same models are validated in the past, where we do not have seasonal labels, we can only examine the global (“any season”) performance. Note that this performance metric cannot be directly compared with the present. Similar positive trends in model performance are found for the 1990–2004 period. Specifically, TSS and AUC_ROC_ increase by +15% and +2.7%, respectively, when comparing M2 to M0 (Figure 2 and Table A3). Overall, M2 achieves the best model performance and more realistically represents the link between different seasonal stages of the migratory cycle, which is confirmed by its ability to better reproduce the niche in the distant past. Therefore, we select the concatenated seasonal model architecture (M2) to project future shifts of the niche of *D. plexippus* under climate change.

**FIGURE 3:**
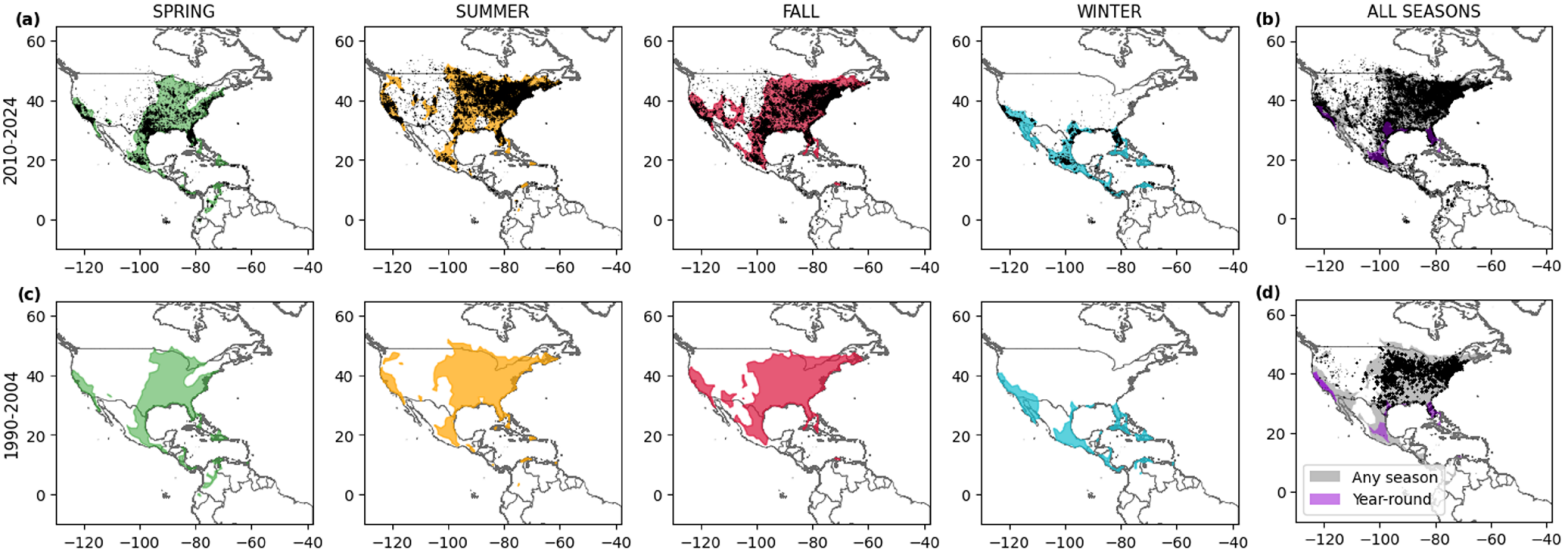
Estimated seasonal (a, c) and global (all seasons, b, d) niches of the monarch butterfly (spring in green, summer in orange, fall in red, winter in cyan, any season in gray, and year‐round in purple) by the concatenated seasonal model (M2) with monarch observations in black in the present (2010–2024, a, b) and past (1990–2004, c, d). Note that the observation records for the time period 1990–2004 lack seasonal information, but we can produce seasonal maps thanks to the M2 model architecture.

Mapping the seasonal distribution of monarchs at a pan‐American scale, we find a good overlap between the estimated niche and the occurrence data (Figure 3). Importantly, the estimated seasonal niches represent well the monarch migratory route, with the summer distribution extending the most northwards and the most concentrated niche in overwintering sites. Our model is able to identify suitable areas that allow monarch presence year‐round, which match well with known areas of stable monarch populations, such as southern California (Satterfield et al. 2016; Urquhart et al. 1970), southern Florida (Brower 1961), the Gulf Coast (Howard et al. 2010), and Mexico (Pfeiler et al. 2017) (Figure 3b,d). When we compared observed and estimated distributions for the year‐round ecological niche of the monarch butterfly, we found a good global overlap between our predictions and the main known locations that host resident populations (Figure A2). However, better‐quality data regarding year‐round monarch locations that do not suffer from partial temporal coverage of seasonal occurrences, paired with a species distribution model targeted to estimating the niche of resident populations, could improve those estimates. Our findings can indeed clarify which climatic conditions favor the stability versus the migratory behavior of monarch butterfly populations. However, our model performance could be improved in the western part of the United States, which does not belong to the estimated niche despite some observations having been recorded.

Compared to the estimated distribution by the seasonal independent model (M1), the concatenated model (M2) seems to be more conservative in predicting suitable areas for the monarch butterfly (Figure A3), which generally translate into similar or higher accuracy across the considered biomes (Figure A4). Some regions at the central, western and southern niche border are indeed excluded by M2, possibly because the previous season probability acts as an additional constraint for monarch suitability. However, at the northern niche border, M2 shows few new suitable areas. The biggest differences between models are found in the fall season, when the entire area of Gulf of California and northern Mexico are classified as unsuitable by M2, suggesting a strong dependency of fall probability on the summer one, when these areas are excluded from the niche. This model difference increases the estimated AUC_ROC_ by 8% in the described regions (Figure A4). Comparing M2 time‐static niche with M0 estimates, the main discrepancies are an underestimation of the niche upper limit at high latitudes and in the south‐western part of the US together with the Gulf of California by M0 (Figure A3), suggesting that annual averages of climatic predictors cannot fully capture the species ecological requirements. The highlighted spatial differences relative to the time‐static niche are relevant and lead to a 7% increase of AUC_ROC_ for M2 compared to M0 (Figure 2).

Ultimately, our time‐aware modeling approach reproduces well the observed occurrences of monarchs, appears to better capture the inter‐seasonal dependency of a continuous migratory movement and more accurately represents monarch suitability niche. Biomes for which M1 performance is higher than M2 are limited and do not translate in significant differences in suitability estimates (Figure A4).

### 3.2 Contribution of Climatic Predictors

**FIGURE 4:**
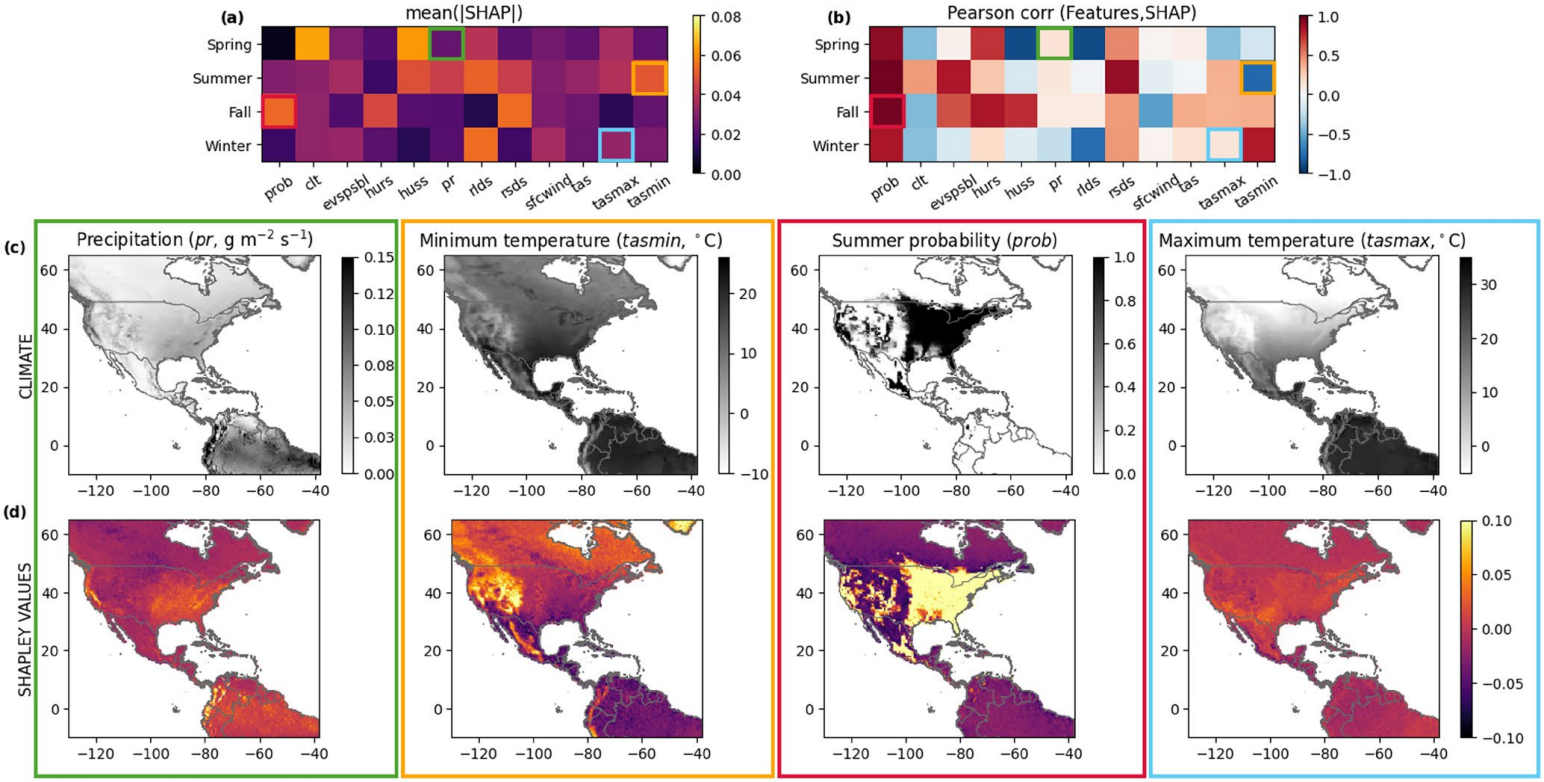
(a) Seasonal mean absolute Shapley values for each climatic predictor and (b) Pearson correlation coefficients between features and Shapley values. Spatial maps of (c) climatic variables and (d) Shapley values of precipitation in spring (*pr*, g m^−2^ s^−1^), minimum temperature in summer (*tasmin*, °C), monarch occurrence probability predicted for summer, when used as a predictor in the fall model (*prob*), and maximum temperature in winter (*tasmax*, °C). The colored boxes highlight the climatic variables that are investigated. Their color represents the considered season: spring (green), summer (orange), fall (red), winter (cyan). Refer to Table A1 for variable abbreviations and full description.

We quantify overall predictor importance using the mean of absolute values of Shapley values (Figure 4a) and estimate the directional effect of the predictors on the species probability of occurrence using Pearson correlation coefficients (Figure 4b). Shapley values allow us to interpret model predictions by examining the concurrent contributions of climatic features to species occurrence in a spatiotemporally‐explicit way. Comparing the importance of each seasonal climatic predictor, relative humidity (*hurs*), downwelling shortwave radiation (*rsds*), and maximum temperature (*tasmax*) seem to be highly positively correlated with Shapley values. Interestingly, the predictor importance varies across seasons, which may result in an inversion of the sign of the correlation coefficient. Overall, cloud coverage and downwelling longwave radiation are more consistently negatively associated with the monarch butterfly occurrence, whereas relative humidity and evaporation are positively related to species presence. As expected, the probability of occurrence of the previous seasons is positively correlated with monarch presence, in particular in summer and fall. This finding confirms that the main differences in the fall niche between M2 and M1 are indeed due to the constraint imposed by a low summer probability in north‐western Mexico (Figure A3), which explains M2's higher performance in the corresponding desert and tropical dry broadleaf forests biomes (Figure A4). Although this positive correlation is also present in the other seasons, it is the weakest in winter, probably due to the drastic shift occurring when reaching the overwintering sites. Temperature‐related predictors (*tas*, *tasmin*, and *tasmax*) positively affect monarch occurrences in fall and winter, but this trend is reversed for spring and summer. These results highlight the complex and multi‐faceted impact of climatic predictors in defining the monarch butterfly occurrence, which becomes even more heterogeneous when we analyze the effect of selected key predictors in a spatially explicit way (Figure 4c,d). In particular, we focus on the Shapley values of precipitation in spring (*pr*), minimum temperature in summer (*tasmin*), monarch occurrence probability predicted for summer, when used as a predictor in the fall model (*prob*), and maximum temperature in winter (*tasmax*). Shapley values of spring precipitation are high in the East and West coasts of the United States, where precipitation is more abundant, compared to the drier central regions. For summer minimum temperature, we have a higher probability of monarch occurrence where minimum temperature is low, such as in mountainous areas belonging to the Rocky Mountains, Sierra Nevada, and Sierra Madres. The importance of the predicted summer distribution for the fall niche is evident, with very high Shapley values corresponding to locations belonging to the summer niche. For winter maximum temperature, we have moderate thermal values, around 15°C, that lead to high Shapley values in the southern part of North America. The different contribution of climatic predictors across seasons and spatial locations highlights the need for season‐aware species distribution models that can be flexibly implemented for the multiple temporal stages of the migration cycle.

### 3.3 Future Shifts in the Monarch Ecological Niche

**FIGURE 5:**
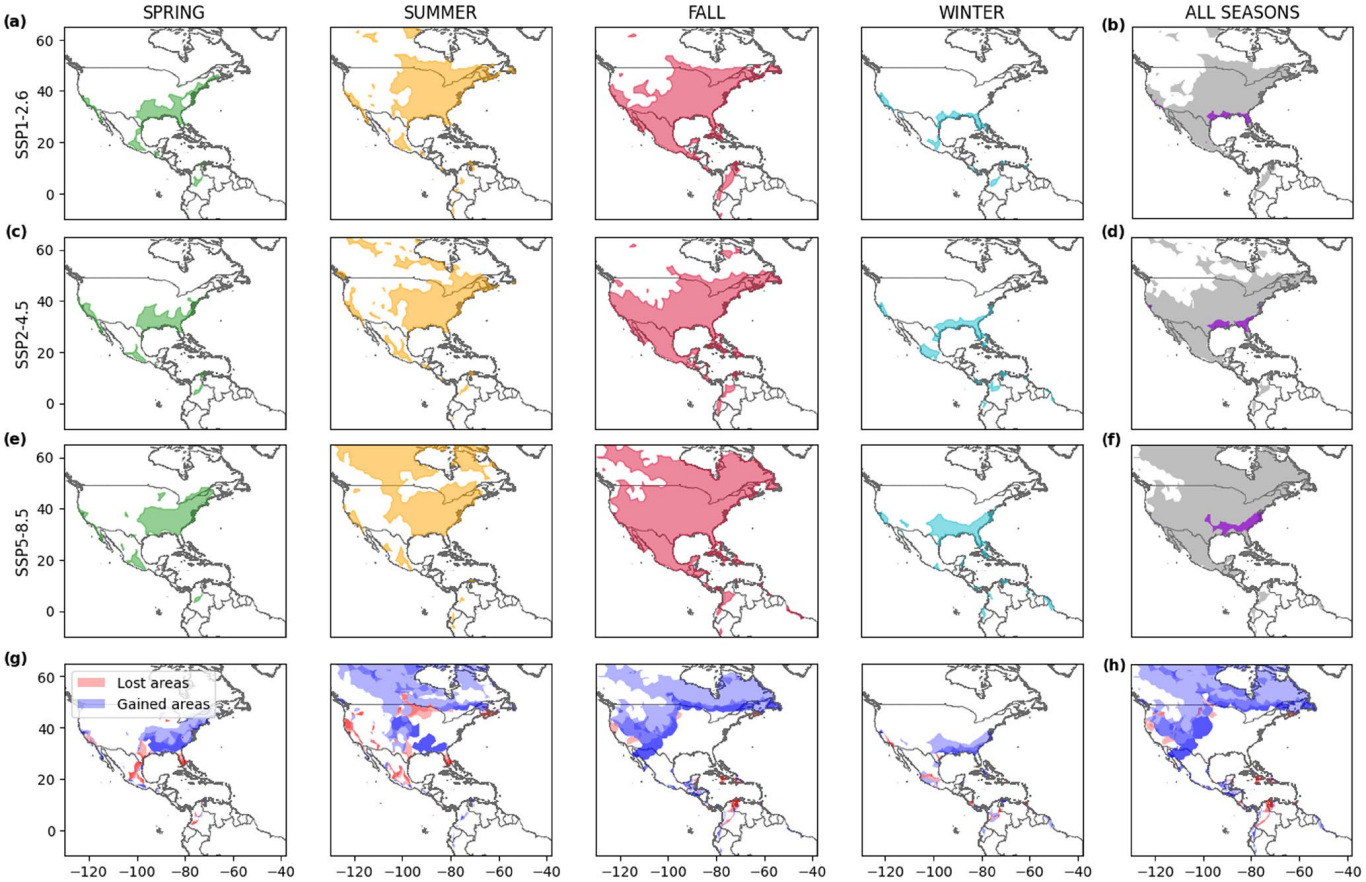
Projected seasonal (a, c, e) and global (all seasons, b, d, f) niches of the monarch butterfly (spring in green, summer in orange, fall in red, winter in cyan, any season in gray, and year‐round in purple) at the end of the XXI century (2086–2100) under SSP1‐2.6 (a, b), SSP2‐4.5 (c, d) and SSP5‐8.5 (e, f) climate change scenarios. Lost (red) and gained (blue) areas, compared to 2010–2024, are represented for each season (g) and for all seasons (h). Darker areas (panels g, h) represent regions of agreement between the three scenarios.

We project future seasonal and season‐independent niches of the monarch butterfly at the middle (Figure A5) and at the end of the XXI century (Figure 5) under multiple climate change scenarios. Going from the mildest (SSP1‐2.6) to the most extreme (SSP5‐8.5) scenario, we notice more evident distributional shifts. Our model predicts a considerable gain in new suitable areas, which is more significant in summer and fall. These new suitable areas would be mainly located on the East Coast for spring, in the northern part of the US and Canada for summer and fall, and in the southern US for winter. In contrast, a contraction of the spring–summer niche in Mexico and in Central America for the fall–winter niche is expected. Overwintering sites would also move northwards, covering up to the entire southern US. These changes in the monarch seasonal distributions can potentially have significant consequences on the suitable areas for year‐round butterfly populations. In this respect, our model predicts the disappearance of the stable populations in Mexico and California, the latter more pronounced under the SSP1‐2.6 (Figure 5a) and SSP5‐8.5 (Figure 5e) scenarios. The Florida resident population is expected to lose its habitat under the SSP5‐8.5 pathway, which represents the scenario with more consistent losses for the year‐round monarch population. These important losses are also predicted for the middle of the century (Figure A5), depicting an alarming situation given that these populations can fail to adapt their resident behavior to a migratory one. Conversely, an expansion of the resident monarch population in the southern states of the United States, such as Texas, Georgia, Louisiana, and Alabama, is projected (Figure 5g). According to the simulations, these regions may represent the only areas where it would be possible to find monarchs year‐round in the future.

**FIGURE 6:**
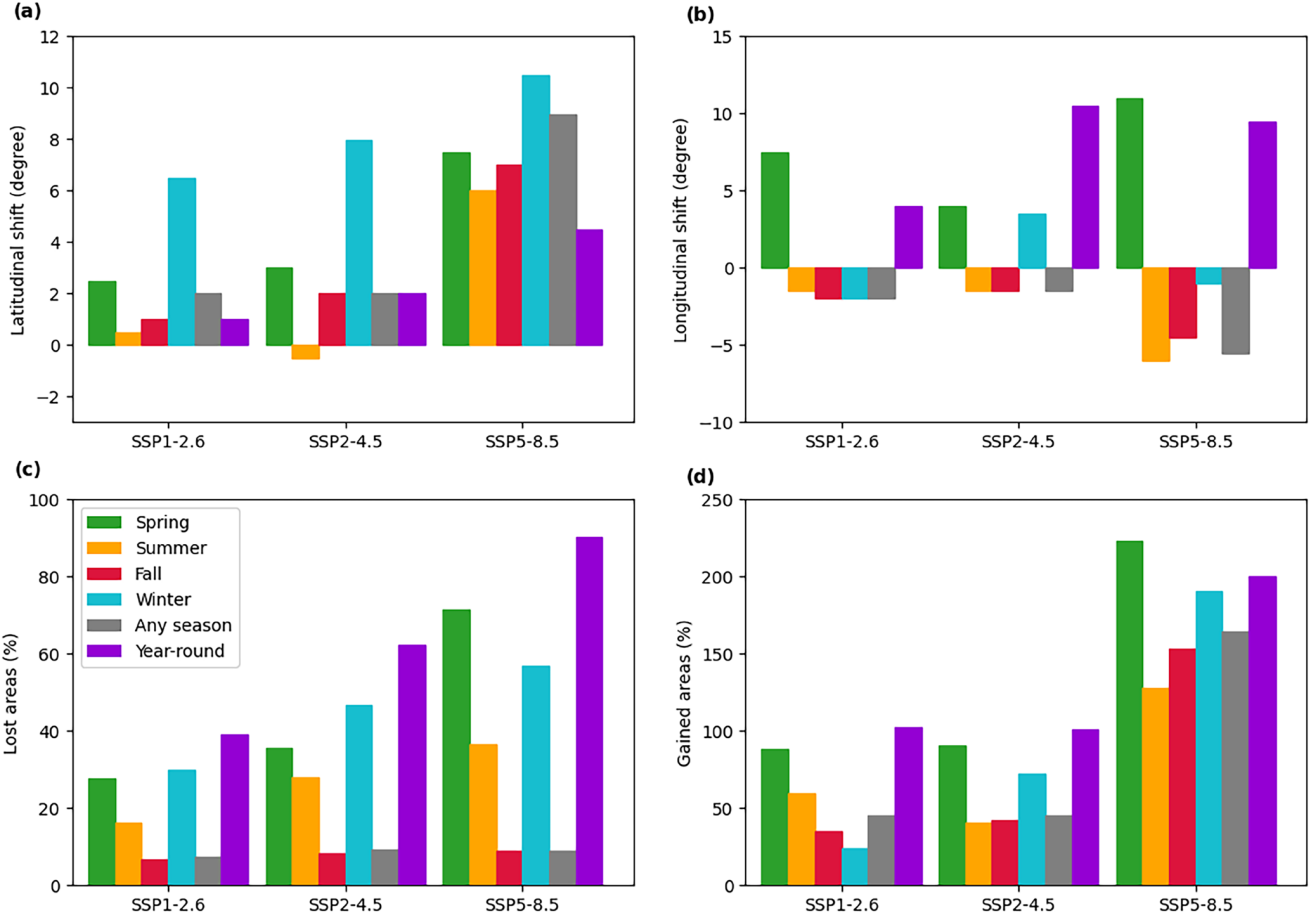
(a) Latitudinal and (b) longitudinal future shifts of the medians of the monarch spatial distribution at the end of the XXI century (2086–2100) under SSP1‐2.6, SSP2‐4.5, and SSP5‐8.5 climate change scenarios compared to the present (2010–2024) for spring (green), summer (orange), fall (red), winter (cyan), any season (gray) and for year‐round (purple). Percentages of (c) lost and (d) gained areas of the monarch butterfly ecological niche are reported. Refer to Figure A6 for detailed information about latitudinal and longitudinal spatial distribution.

Analyzing the latitudinal and longitudinal shifts of the monarch ecological niche (Figure 6a,b and Figure A6), we expect a median northward shift of +2° or +9° and a western shift of −2° or −5.5° for SSP1‐2.6 or SSP5‐8.5, respectively. The most prominent latitudinal shifts are predicted for the upper limits of the summer and fall niches, with a maximum of +16° and +13° of the 95th percentile, respectively (Figure A6). Longitudinal eastern shifts of the spring distribution, due to the contraction and loss of suitable areas on the West Coast, would result in the same directional shift of the year‐round niche. The highlighted climate‐driven changes would result in the loss of suitable areas for monarchs, ranging from 10% to 90% of the current niche, compared to the present (Figure 6c). Although the estimated extension of niche expansion areas outnumbers the lost ones (Figure 6d), the ability of monarchs to reach, adapt, and thrive in those new areas remains unknown.

**FIGURE 7:**
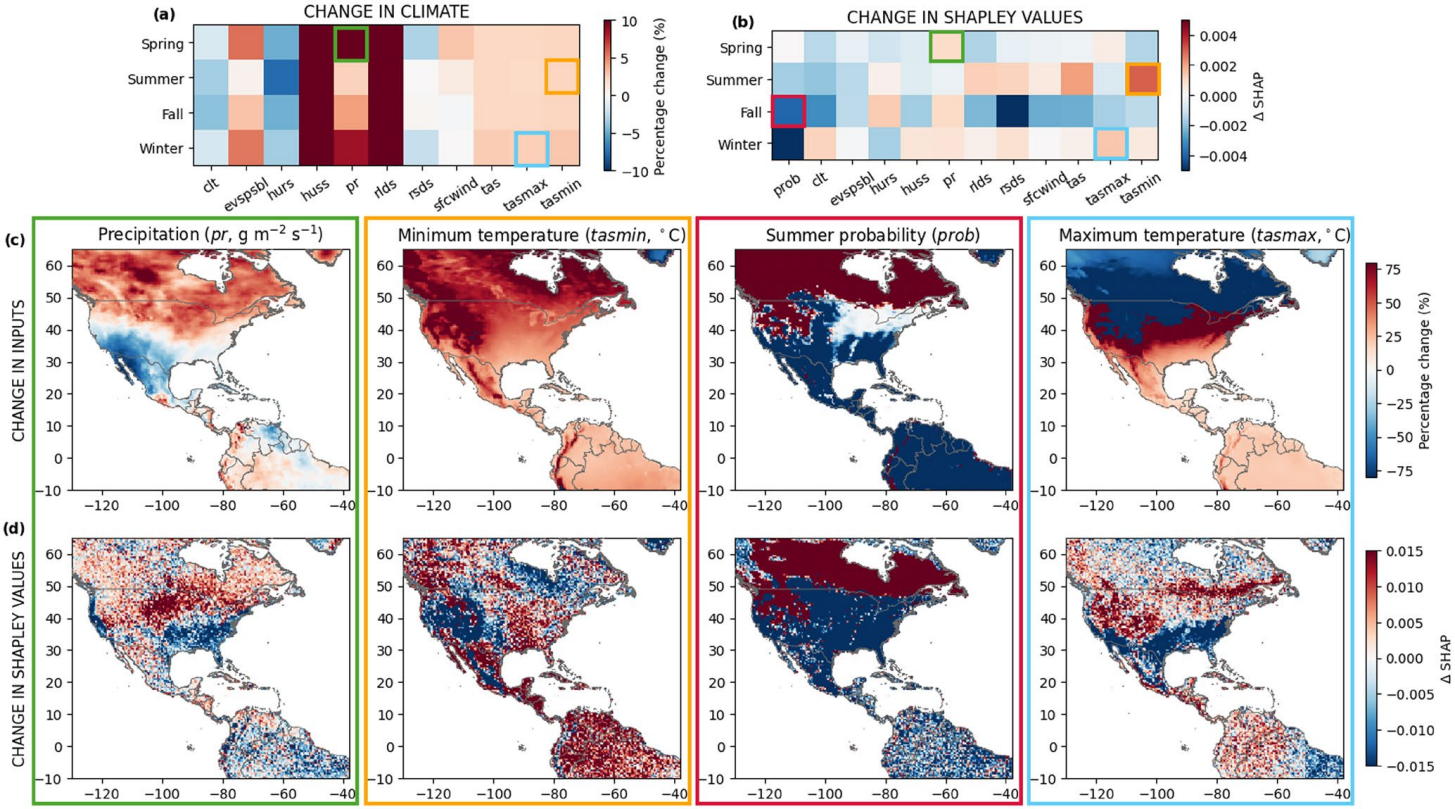
Change in (a) climatic variables and (b) Shapley values between the SSP5‐8.5 scenario at the end of the XXI century (2086–2100) and the historical baseline (2010–2024) for each predictor and season. Spatial maps of (c) percentage input changes and Δ changes in Shapley values of precipitation in spring (*pr*, g m^−2^ s^−1^), minimum temperature in summer (*tasmin*, °C), probability of occurrence in summer for the fall distribution (*prob*), and maximum temperature in winter (*tasmax*, °C), compared to 2010–2024. The colored boxes highlight the climatic variables that are investigated. Their color represents the considered seasons: spring (green), summer (orange), fall (red), winter (cyan). Refer to Table A1 for variable abbreviations and full description.

Thanks to the implementation of the concatenated seasonal deep SDM modeling approach, not only can we visualize the expected future niche shifts described above, but also determine which changes in climatic drivers are responsible for producing these patterns. Computing the average difference between Shapley values in the future compared to the selected historical baseline (2010–2024), it becomes possible to disentangle the role of the multiple model predictors in determining the niche shifts (Figure 6). At the end of the century, under the SSP5‐8.5 scenario, a positive difference in Shapley values is found for temperature predictors in winter, indicating a higher suitability for monarchs, probably due to less restrictive thermal conditions. Evapotranspiration and specific humidity are generally expected to be less favorable for monarch occurrence, in particular in summer and fall (Figure 6b). Less prominent, but similar trends across the three considered scenarios are expected at the middle of the century (Figure A7). Exploring changes in Shapley values spatially (Figure 7c,d), we find increased precipitation in spring in the north‐central United States. Conversely, the opposite trend would occur due to drier conditions in California and the Gulf Coast. A similar and more prominent pattern is expected for the winter niche due to higher maximum temperatures. These latter changes, together with the combination of the other climatic predictors, are responsible for the future contraction of the year‐round niche of monarch butterflies. A drastic increase in minimum temperature in summer in the mountainous regions is associated with a decrease in Shapley value and lower suitability for the monarch butterflies. Importantly, these seasonal changes would have implications on the other seasons. For instance, we highlight how shifts in monarch summer distribution could impact fall Shapley values and consequently species suitability. The described trends are expected to be milder under SSP1‐2.6 and SSP2‐4.5 (Figures A8 and A9).

## 4 Discussion

How is climate change likely to alter species ranges and migration patterns? Which environmental variables and climate change scenarios would be responsible for detrimental impacts on the ecology of migratory populations and the key ecosystem services they provide? In this study, we develop a season‐aware deep learning approach to model the ecological niche of an iconic migratory species, the monarch butterfly ( *D. plexippus* ), at a pan‐American scale. Specifically, our seasonal model can accurately reproduce the full migratory cycle of monarchs across seasons. This framework not only shows better performance than the conventional time static approach on our held‐out test set, but also outperforms it when estimating any season niche from historical data from different climatic conditions compared to those considered in model training. Importantly, we have shown how the integration of the previous season estimates into the concatenated modeling framework can constrain suitable areas and better capture the temporal dependence of the migratory movement across different biomes. Finally, we predict future shifts of the monarch niche under multiple climate change scenarios. Using Shapley values, an explainable AI method, we establish the contribution of each climatic predictor in shaping *D. plexippus* current and future ecological niche at the global scale in a spatially explicit way.

Our findings suggest that humidity, temperature, and precipitation, in addition to cloud coverage and shortwave radiation, play an important role in shaping the ecological niche of *D. plexippus* . The relevance of these climatic variables for the monarch population size and distribution is in agreement with previous literature (Zylstra et al. 2021; Zipkin et al. 2012; Oberhauser and Peterson 2003). The climatic effects that we highlighted vary in space and in time, with contrasting contributions across the study region and in the different months. Importantly, the contribution of climatic predictors to the winter ecological niche is very distinct from the rest of the year. Given the environmental sensitivity of the wintering diapause state (Masters et al. 1988; Green and Kronforst 2019), mapping the climatic suitability of overwintering sites is of high interest for butterfly conservation and for long‐term sustainability of the host population (Fisher et al. 2024). At the end of the XXI century, our findings suggest a north‐western shift of the monarch niche of [+2; +9] degrees in latitude and [−2; −5.5] degrees in longitude, depending on the realized climate change scenario. These distributional changes would be the result of a consistent expansion of suitable areas situated at the northern border of the seasonal ecological niche, counterbalanced by the niche contraction in the southern regions. These losses are expected to involve more than half of the regions currently occupied by resident monarch populations and are mainly located in California and Mexico. The latter regions are notable for being the main monarch winter diapause site and a main attraction for ecotourism (Barkin 2003). Their loss would indeed constitute not only a critical drawback for the Eastern monarch population, but also a main disadvantage for Mexican biodiversity and economy. The exclusion of these crucial areas from the monarch niche could have disruptive consequences for the population and migratory cycle (Zylstra et al. 2021; Flockhart et al. 2017). These considerations regarding predicted range shifts in the monarch niche, in particular for breeding and overwintering grounds, provide essential insights into the monarch migration that should be considered by conservation planning (Grant and Bradbury 2019).

The study of climate change impacts on species ecological niche faces numerous challenges due to the complex species‐specific responses and the simultaneous effect of multiple climatic features. For instance, counteractive climate change impacts on species ranges have already been documented (Lenoir et al. 2010; Crimmins et al. 2011), such as downslope shifts, highlighting the substantial heterogeneity in the climate change effects on species distribution (Rubenstein et al. 2023). The study of the ecological niche of migratory species exacerbates the complexity of this issue by adding the temporal component to the niche study (Ponti and Sannolo 2023). This requires adequate modeling techniques that can disentangle the importance of the multiple climatic effects on species niche in a spatio‐temporally dependent manner. The implementation of XAI methods fulfills this need by identifying the relevant predictors linked to the species occurrence in different seasons and responsible for species range shifts (Zbinden et al. 2025; Bourhis et al. 2023). In our work, we use Shapley values to achieve this task (Lundberg and Lee 2017; Shapley 1953), and our findings highlight that the same climatic change can have contrasting consequences across the spatial domain and the four seasons. Overall, XAI represents a powerful resource to bridge the gap between ecological relevance for species distribution and black‐box modeling tools (Ryo et al. 2021). Examining changes in Shapley values under different climatic scenarios, we identify whether and which climatic variables would be responsible for the predicted shifts, both globally and locally, for the monarch butterfly niche. These findings should be interpreted with consideration of their limitations. Our modeling approach cannot detect a potential temporal misalignment that might exist between the environmental suitability and monarch occurrence. For instance, monarchs could leave an area that still belongs to its suitable range, or vice versa, they could stay in an area for a period of time even if its conditions have become unsuitable. Our modeling framework is based on recorded monarch presences and identifies the realized ecological niche of the monarch butterfly, that is, the actual set of conditions that a species occupies (Hutchinson 1957). To further study the possibility of a temporal misalignment, a more ecological‐mechanistic approach could be used to compare the fundamental range, where the species could potentially survive, with the actual range identified by our model for each analyzed temporal interval. The highlighted issue could become even more critical when shorter temporal intervals are examined, for example, months or weeks rather than seasons, and could produce mismatched niche‐environment mapping. Although bringing novel insights into the biogeography of migratory species under climate change, our study is not exempt from limitations. Statistical modeling frameworks for species distribution have a limited predictive capability in very different climatic settings (Santini et al. 2021). Although we test that our approach works well for past climatic conditions, this does not exclude inaccuracies when the most extreme climatic scenarios are examined. Importantly, our considerations are based on the monarch response to current and past environmental conditions, lacking the inclusion of potential evolutionary dynamics that would drive adaptation to future climate. In addition, true species absences should be used in model training and validation, although those types of data are limited compared to presence‐only observations from which we derived background points (Whitford et al. 2024). While we explicitly incorporate the intra‐annual (seasonal) variability of climatic variables and the temporal sequence of seasonal niches, the climatic inter‐annual differences within the considered temporal intervals are neglected. A possible way to integrate yearly dynamics could be to implement our seasonal model architecture year by year and to obtain a more precise niche estimate over time. However, this annual analysis would be more suited to short‐term forecasting rather than long‐term estimates of climate change impacts. In addition to inter‐annual climate variability, the inclusion of additional bioclimatic predictors could potentially improve our model performance.

Our modeling approach advances current knowledge on the species' climatic suitability, but needs to be paired with a population ecological model to investigate the effective impacts of climate change on monarch population abundance and survival. In addition, our framework considers the effect of multiple climatic features on the distribution of monarch butterflies, disregarding biotic interspecific ecological interactions. *D. plexippus* larvae feed on specific host plants, that is, milkweeds (*Asclepias* spp.) (Dilts et al. 2019), whose population is experiencing a widespread decline due to the intensive use of glyphosate herbicides for agriculture (Zylstra et al. 2021). The decreasing abundance of native milkweed threatens the monarch population to be captured in a trophic ecological trap: the non‐native and invasive milkweed ( *Asclepias curassavica* ) is increasing the sedentary behavior of the monarch population by providing a year‐round food source, but of lower quality that can negatively impact butterfly mass and survival (Faldyn et al. 2018). Therefore, monarch ecological niche shifts under global warming should be evaluated in relation to milkweeds, natural enemies (McCoshum et al. 2016) and infectious diseases, such as the devastating protozoa *Ophryocystis elektroscirrha*. Deep learning approaches like ours represent a flexible and valid option to integrate these multi‐trophic interactions and their potential effects on monarch distribution. We leave these studies for future work.

Although the monarch butterfly presents a unique migration pattern and climatic dependencies specific to its population ecology, it is not the only species that needs to travel through a warming world: migration is a widespread behavior in the animal kingdom, in particular for the insect group, the most species‐rich of terrestrial migrants (Chapman et al. 2015), with more than 600 migratory species only for butterflies (Chowdhury et al. 2021). The developed modeling framework can indeed be applied to these other species to study their distributional shifts during their migration journey. More specifically, our approach is more effectively transferable to species that present a gradual seasonal movement, which can be more effectively captured by the concatenated architecture of the proposed model, rather than a fast migration that creates spatially separated seasonal niches. In the latter case, a narrower temporal window, such as a monthly time step, could be implemented to better capture short migratory pulses. Beyond migration, our time‐aware SDM can also be implemented to other seasonal distributional shifts, as the altitudinal movement along the elevation gradient of mountainous species. In all the mentioned cases, model transferability requires (i) an adequate spatiotemporal coverage of species occurrence data to properly train the model, although we showed that data‐deficient time windows can borrow strength from more intensively sampled periods, (ii) the inclusion of time‐varying environmental predictors that are linked to species distributional shifts and their ecological requirements, and (iii) a resolution of both species and environmental datasets that matches the scale of applicability of the specific study (e.g., global, regional, or local). A limitation of the current setup is that individuals are not tracked along the seasons, as there is no individual identification of single butterflies in the GBIF dataset. In other words, we map the niches, but we are unable to analyze the journey of specific populations. Tracking data would indeed be needed to move toward a more fine grained analysis of migration patterns.

Ultimately, our approach holds the potential to advance scientific understanding regarding the ecological niche of other strongly seasonal taxa and their vulnerability to global warming. Investigating the current and future challenges that climate change poses to species macroecology and biogeography is essential to implement effective conservation strategies aimed at preserving biodiversity and ecosystem health.

## Author Contributions

**Chiara Vanalli:** conceptualization, data curation, formal analysis, investigation, methodology, validation, visualization, writing – original draft, writing – review and editing. **Robin Zbinden:** investigation, methodology, writing – review and editing. **Nina van Tiel:** investigation, methodology, writing – review and editing. **Devis Tuia:** conceptualization, funding acquisition, investigation, methodology, supervision, validation, writing – original draft, writing – review and editing.

## Funding

This work was supported by Schweizerischer Nationalfonds zur Förderung der Wissenschaftlichen Forschung (204057).

## Conflicts of Interest

The authors declare no conflicts of interest.

## Supporting information


Data S1.

## Data Availability

The data that support the findings of this study are openly available in GBIF at https://doi.org/10.15468/dl.repydp (23 September 2024). The data and code that support the findings of this study are openly available in a Figshare repository at https://doi.org/10.6084/m9.figshare.29468831.
