# Supplementary material for: Migrating in a Warming World: A Deep Learning Approach to Predict Pan‐American Seasonal Shifts in the Monarch Butterfly Niche
Source: Glob Chang Biol. 2026 Mar 27;32(3):e70805. doi: 10.1111/gcb.70805 (PMC13022812; doi:10.1111/gcb.70805)
Supplement: Supplementary file 1 — Data S1. [file GCB-32-e70805-s001.pdf]

Migrating in a warming world: A deep learning approach to predict pan-American seasonal shifts in the monarch butterfly niche

Chiara Vanalli[1*], Robin Zbinden[1], Nina van Tiel[1], and Devis Tuia[1*]

[1]Environmental Computational Science and Earth Observation Laboratory, École Polytechnique Fédérale de Lausanne, Sion 1950, Switzerland
[*]Corresponding authors: chiara.vanalli@epfl.ch, devis.tuia@epfl.ch

Supplementary Information

Table A1: Climatic predictors, short names and unit of measure

**Climatic variables**	**Short name**	**Unit**
Near-surface mean air temperature	tas	K
Near-surface min air temperature	tasmin	K
Near-surface max air temperature	tasmax	K
Near-surface specific humidity	huss	-
Near-surface relative humidity	hurs	%
Evaporation including sublimation and transpiration	evspsbl	Kg m^{-2} s^{-1}
Surface downwelling shortwave radiation	rsds	W m^{-2}
Surface downwelling longwave radiation	rlds	W m^{-2}
Near-surface wind speed	sfcwind	m s^{-1}
Precipitation	pr	Kg m^{-2} s^{-1}
Total cloud coverage	clt	-

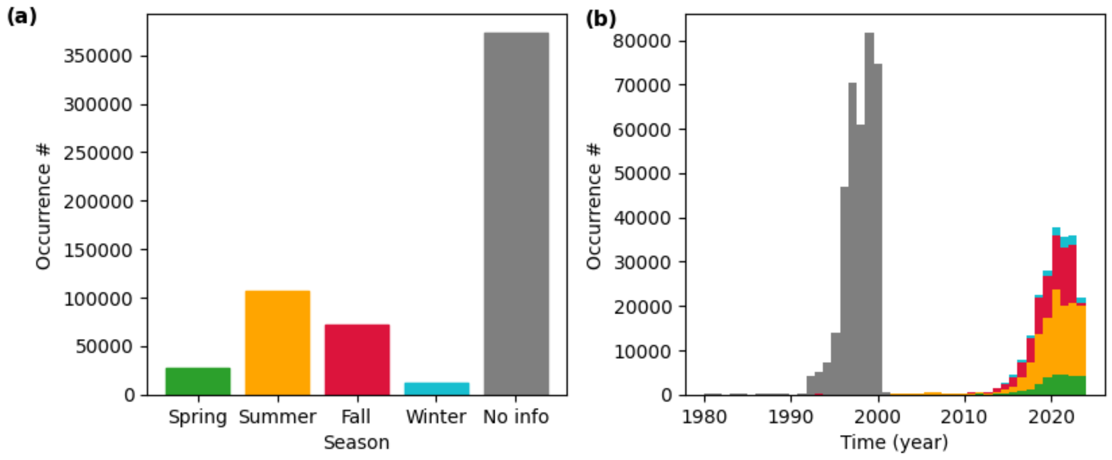

Figure A1: Monarch butterfly occurrence data: (a) total number of observations and (b) distribution in time of available observations in spring (green), summer (orange), fall (red), winter (cyan), and for samples without seasonal information (gray).

Table A2: Global (any season) and seasonal model performance comparison of the Area Under the ROC Curve ($\mathrm{AUC_{ROC}}$), the Area Under the Precision-Recall Curve ($\mathrm{AUC_{PR}}$), and the True Skill Statistic (TSS) using the test dataset of 2010-2024. M0 represents the time-static model, M1 the seasonal independent model, and M2 the seasonal concatenated model, respectively. The percentage increase in model performance compared to the time-static model M0 is reported in square brackets.

	Model	AUC_{ROC}	AUC_{PR}	TSS
Global	M0	0.881	0.930	0.683
	M1	0.935 [+6.1%]	0.974 [+4.7%]	0.798 [+16.8%]
	M2	0.944 [+7.0%]	0.978 [+5.2%]	0.805 [+17.9%]
Spring	M0	0.875	0.927	0.674
	M1	0.945 [+8.0%]	0.972 [+4.9%]	0.806 [+19.6%]
	M2	0.951 [+8.7%]	0.975 [+5.2%]	0.811 [+20.3%]
Summer	M0	0.898	0.979	0.716
	M1	0.934 [+4.0%]	0.989 [+1.0%]	0.766 [+7.0%]
	M2	0.942 [+4.9%]	0.991 [+1.2%]	0.783 [+9.4%]
Fall	M0	0.888	0.971	0.684
	M1	0.910 [+2.5%]	0.978 [+0.7%]	0.758 [+10.8%]
	M2	0.914 [+4.0%]	0.977 [+0.6%]	0.764 [+11.7%]
Winter	M0	0.862	0.843	0.659
	M1	0.951[+10.3%]	0.955 [+13.3%]	0.861 [+30.7%]
	M2	0.966 [+12.1%]	0.967 [+14.7%]	0.860 [+30.5%]

Table A3: Global (any season) model performance comparison of the Area Under the ROC Curve (AUC_{ROC}), the Area Under the Precision-Recall Curve (AUC_{PR}) and the True Skill Statistic (TSS) using the time-static dataset of 1990-2004. M0 represents the time-static model, M1 the seasonal independent model, and M2 is the seasonal concatenated model, respectively. Percentage increase in model performance compared to the time-static model M0 is reported in square brackets.

	Model	AUC_{ROC}	AUC_{PR}	TSS
Global	M0	0.874	0.992	0.612
	M1	0.882 [+0.9%]	0.992 [+0%]	0.695 [+13.6%]
	M2	0.898 [+2.7%]	0.993 [+0.1%]	0.713 [+15%]

Table A4: Global (any season) and seasonal MaxEnt (linear, hinge and product feature classes) model performance with both of the Area Under the ROC Curve (AUC_{ROC}), the Area Under the Precision-Recall Curve (AUC_{PR}), and the True Skill Statistic (TSS) using the test dataset of 2010-2024. M0 performance is reported for comparison.

Model	Performance	AUC_{ROC}	AUC_{PR}	TSS
MaxEnt	Global	0.880	0.934	0.660
	Spring	0.880	0.926	0.685
	Summer	0.883	0.981	0.657
	Fall	0.889	0.974	0.666
	Winter	0.866	0.853	0.632
M0	Global	0.881	0.930	0.683
	Spring	0.875	0.927	0.674
	Summer	0.898	0.979	0.716
	Fall	0.888	0.971	0.684
	Winter	0.862	0.843	0.659

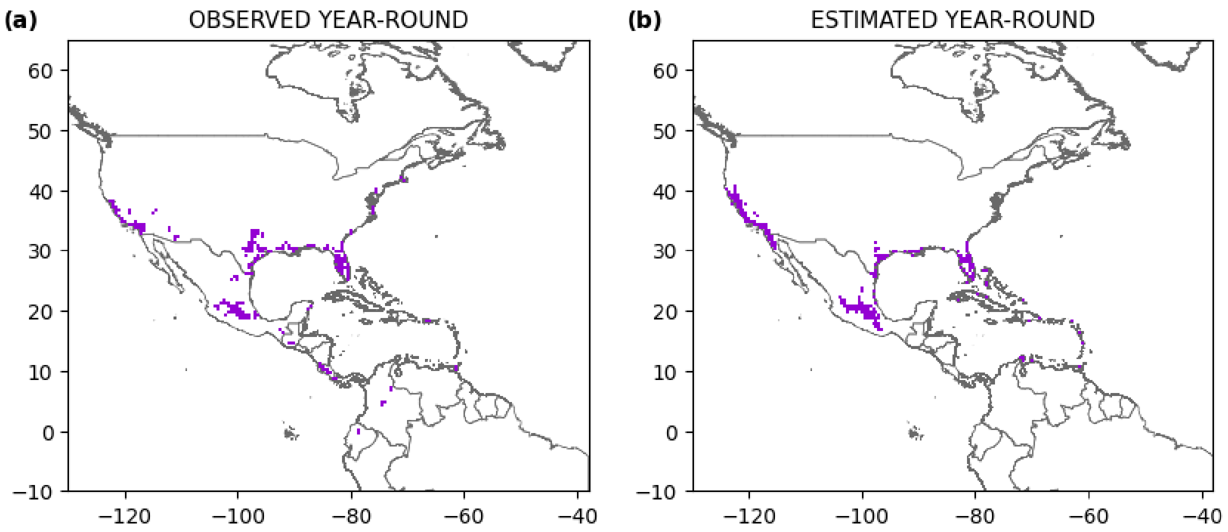

Figure A2: Year-around distributions for the Monarch butterfly. (a) Observed distribution and (b) estimated distribution with the seasonal concatenated model (M2). Both panels illustrate the present period (2010-2024), with suitable locations represented in purple.

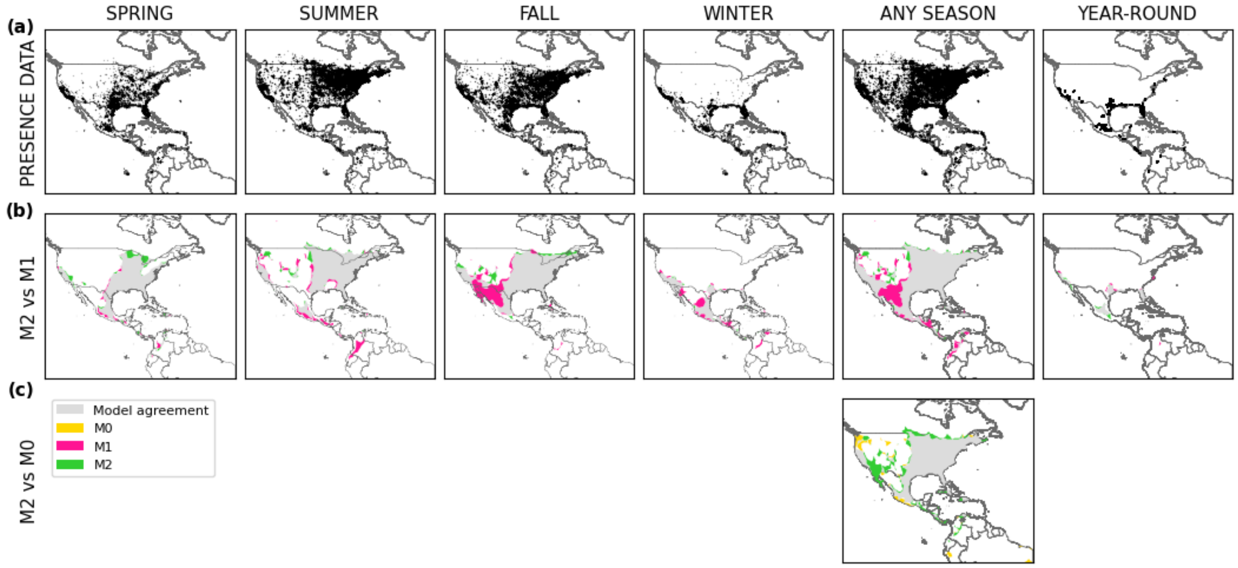

Figure A3: Model comparison of M0 (time-static), M1 (seasonal independent), and M2 (seasonal concatenated) suitability areas for spring, summer, fall, winter, any season and year-round in the period 2010-2024. (a) Presence data, (b) comparison between M2 and M1, (c) comparison between M2 and M0, with overlapping areas of model agreement for species suitability represented in gray, M0-only suitable areas in yellow, M1-only suitable areas in pink, and M2-only suitable areas in green. Note that with M0, it is only possible to estimate the time-static niche and seasonal/year-round comparisons are not possible.

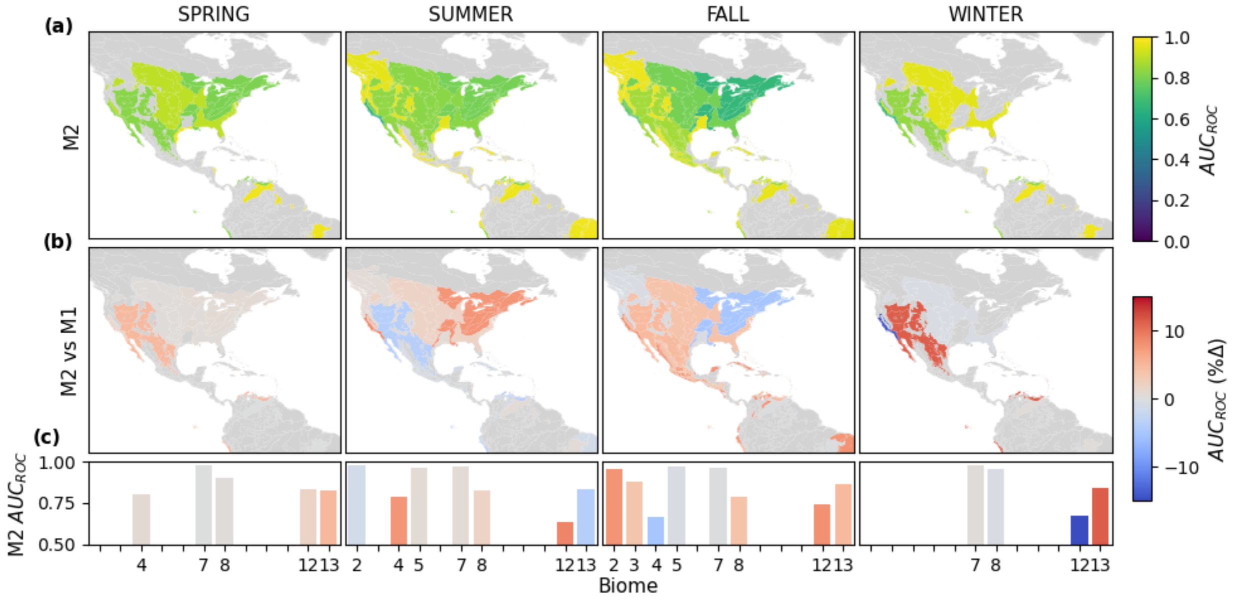

Figure A4: Model comparison of M1 (seasonal independent) and M2 (seasonal concatenated) model accuracy, according to AUC_{ROC} calculated for the test dataset, across different biomes. (a) AUC_{ROC} of M2 (from blue to yellow), (b) percentage difference of AUC_{ROC} between M2 and M1 (from blue to red), (c) histogram of AUC_{ROC} performance of M2 for different biomes; each bar is colored according to the AUC_{ROC} percentage difference with M1. Biome 2=Tropical & subtropical dry broadleaf forests, biome 3=Tropical & subtropical coniferous forests, biome 4=Temperate broadleaf & mixed forests, biome 5=Temperate conifer forests, biome 7=Tropical & subtropical grasslands, savannas & shrublands, biome 8=Temperate grasslands, savannas & shrublands, biome 12=Mediterranean forests, woodlands & scrublands, biome 13=Deserts & Xeric shrublands.

Algorithm 1 Training algorithm for the seasonal concatenated model (M2)

```
for each epoch e do
    Randomly sample starting season;
    Define seasons sequence S as the chronological sequence of the four seasons;
    Organize input data by season, with x_s = [x_s^tr, x_s^val], ∀s ∈ S;
    Set starting dummy input values: d_1 = 0 and d_{2,3,4} = 1;
    Set probability of starting season s as p_0 = 0.5;
    for each season s ∈ S do
        Select training and validation samples for season s: x_s = [x_s^tr, x_s^val];
        Append additional inputs to input seasonal data: x'_s = [x_s, d_s, p_{s-1}];
        Chain forward passes from the first season to season s to obtain prediction ŷ_s;
        Update model weights w_s and set probability of season s as p_s = ŷ_s;
        Compute seasonal cross-entropy loss on validation data Loss_s(x_s^val);
    end for
    Compute global cross-entropy loss on validation data, weighting the season-specific losses to the
    inverse of sample size for each season n_s: LOSS^val = Σ_{s=1}^4 (1/n_s) Loss_s(x_s^val);
end for
Output Select epoch that minimizes the global cross-entropy loss LOSS^val and retrieve the relative
    seasonal trained models w_s together with species occurrence predictions ŷ_s.
```

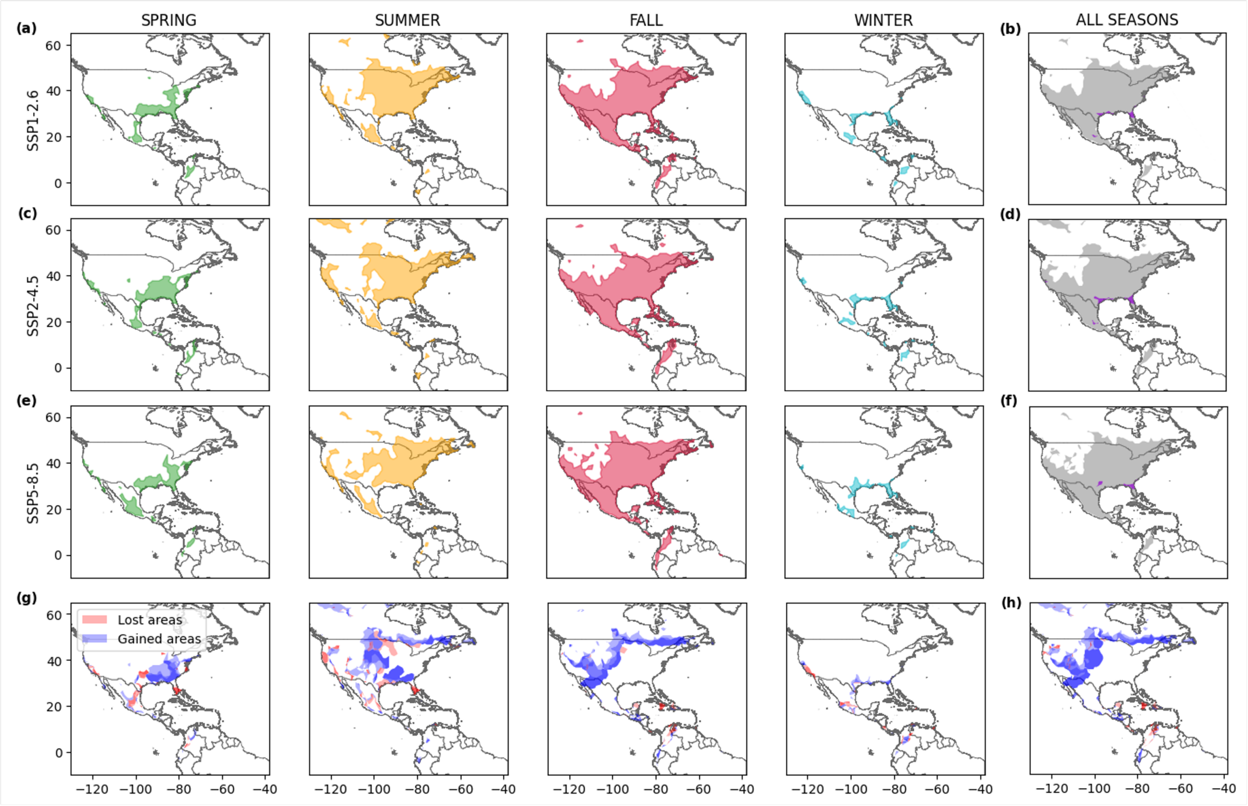

Figure A5: Projected seasonal seasonal (a, c, e) and global (all seasons, b, d, f) niches of the monarch butterfly (spring in green, summer in orange, fall in red, winter in cyan, any season in gray and year-round in purple) at the middle of the XXI century (**2040-2054**) under SSP1-2.6 (a, b), SSP2-4.5 (c, d) and SSP5-8.5 (e, f) climate change scenarios. Lost (red) and gained (blue) areas, compared to 2010-2024, are represented for each season (g) and for all seasons (h). Darker areas (panels g, h) represent regions of agreement between the three scenarios.

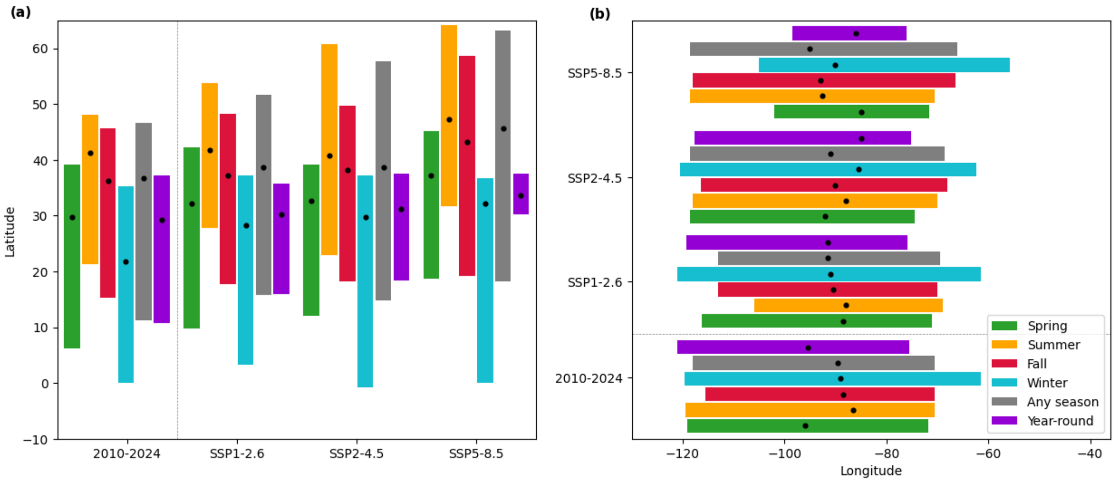

Figure A6: Latitudinal (a) and longitudinal (b) distribution of the monarch butterfly niche (spring in green, summer in orange, fall in red, winter in cyan, any season in gray and year-round in purple) in 2010-2024 and at the end of the XXI century (2086-2100) under SSP1-2.6, SSP2-4.5 and SSP5-8.5 climate change scenarios. The bars are plotted within the 5th and the 95th percentile of latitude and longitude, while the black dots represent the medians.

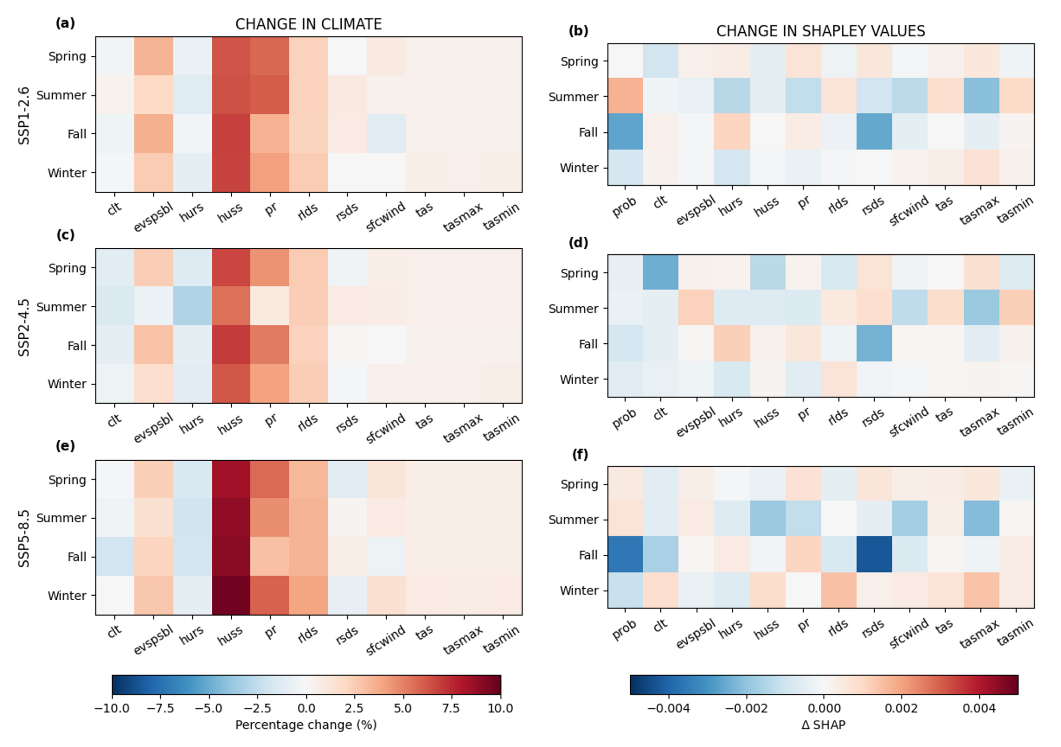

Figure A7: Change in (a, c, e) climatic variables and (b, d, f) Shapley values between the SSP1-2.6 (a, b), SSP2-4.5 (c, d), and SSP5-8.5 (e, f) scenario at the middle of the XXI century (**2040-2054**) and the historical baseline (2010-2024) for each predictor and season.

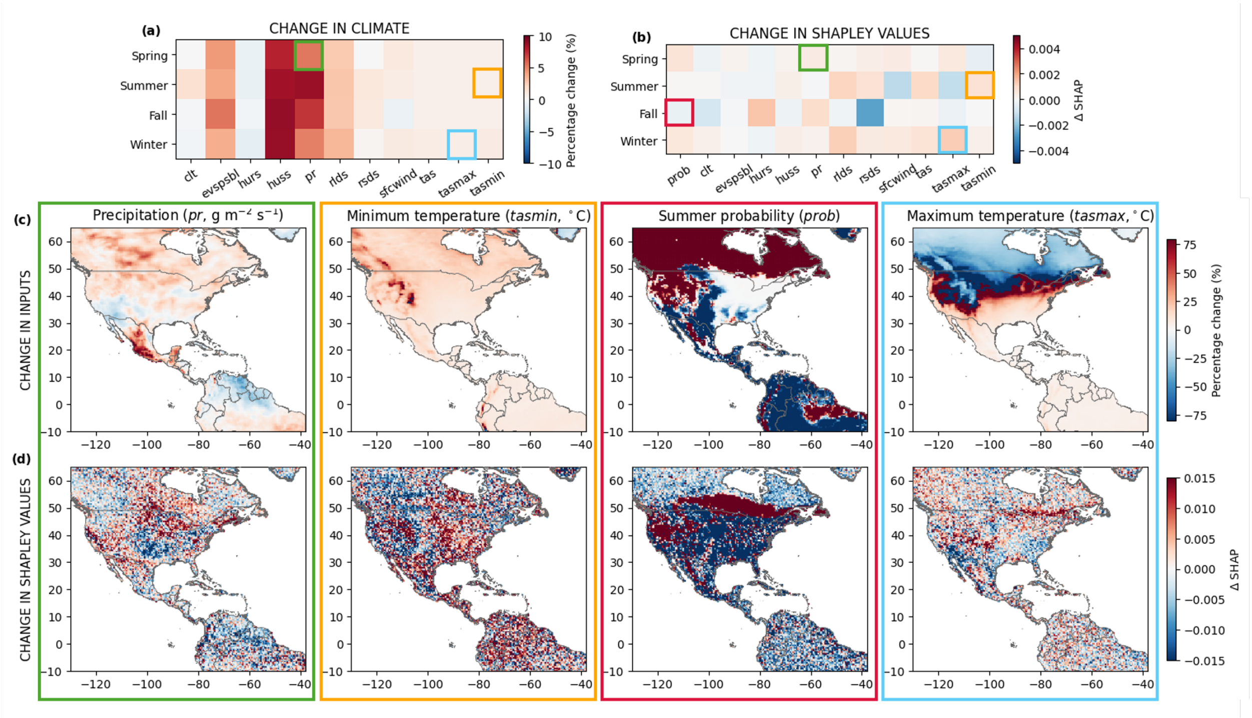

Figure A8: Change in (a) climatic variables and (b) Shapley values between the **SSP1-2.6** scenario at the end of the XXI century (2086-2100) and the historical baseline (2010-2024) for each predictor and season. Spatial maps of (c) percentage input changes and Δ changes in Shapley values of precipitation in spring (*pr*, g m^{-2} s^{-1}), minimum temperature in summer (*tasmin*, °C), probability of occurrence in summer for the fall distribution (*prob*), and maximum temperature in winter (*tasmax*, °C), compared to 2010-2024. The colored boxes highlight the climatic variables that are investigated. Their color represents the considered seasons: spring (green), summer (orange), fall (red), winter (cyan). Refer to Table A1 for variable abbreviations and full description.

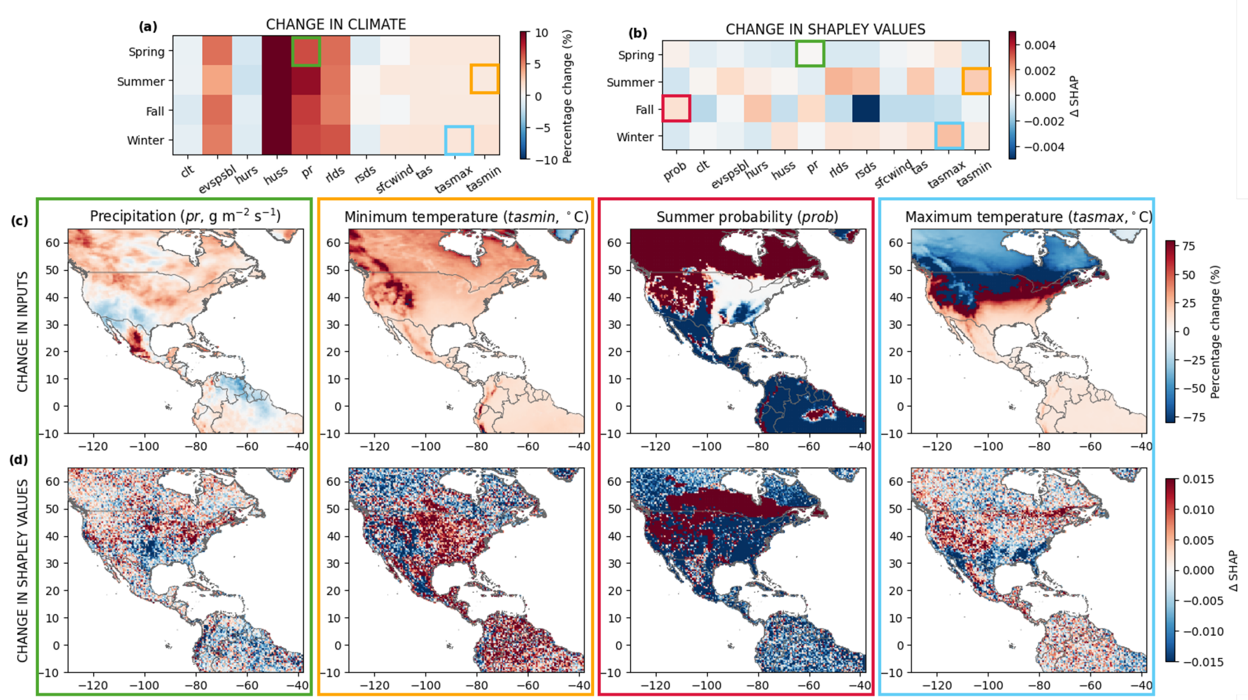

Figure A9: Change in (a) climatic variables and (b) Shapley values between the **SSP2-4.5** scenario at the end of the XXI century (2086-2100) and the historical baseline (2010-2024) for each predictor and season. Spatial maps of (c) percentage input changes and Δ changes in Shapley values of precipitation in spring (*pr*, g m^{-2} s^{-1}), minimum temperature in summer (*tasmin*, °C), probability of occurrence in summer for the fall distribution (*prob*), and maximum temperature in winter (*tasmax*, °C), compared to 2010-2024. The colored boxes highlight the climatic variables that are investigated. Their color represents the considered seasons: spring (green), summer (orange), fall (red), winter (cyan). Refer to Table A1 for variable abbreviations and full description.
